# The role of RENAL, PADUA, C-index, CSA nephrometry systems in predicting ipsilateral renal function after partial nephrectomy

**DOI:** 10.1186/s12894-019-0504-2

**Published:** 2019-08-05

**Authors:** Yu-De Wang, Chi-Ping Huang, Chao-Hsiang Chang, Hsi-Chin Wu, Che-Rei Yang, Yu-Ping Wang, Po-Fan Hsieh

**Affiliations:** 1 Department of Urology, China Medical University Hospital, No. 2, Yu-De Rd., Taichung City, 404 Taiwan, Republic of China; 2 School of Medicine, China Medical University, No. 91, Xueshi Rd., North Dist, Taichung City, 404 Taiwan, Republic of China; 3 Department of Urology, China Medical University Beigang Hospital, No. 123, Xinde Rd., Beigang Township, Yunlin County, 651 Taiwan, Republic of China; 4 Department of Radiology, Taichung Veterans General Hospital, No. 1650, Sec. 4, Taiwan Blvd., Xitun Dist., Taichung City, 407 Taiwan, Republic of China

**Keywords:** Contact surface area, Partial nephrectomy, Renal function, Nephrometry, Renal scintigraphy

## Abstract

**Background:**

Functional outcome is an important issue in nephron-sparing surgery. Various nephrometries have been developed to predict renal function preservation. The aim of this study was to examine the applicability of R.E.N.A.L., PADUA, C-index, and mathematical tumor contact surface area (CSA) in predicting ipsilateral renal function after partial nephrectomy using radio-isotope scans.

**Methods:**

We performed this retrospective study in patients who underwent partial nephrectomy between May 2013 and April 2017, and used abdominopelvic computerized tomography or magnetic resonance imaging to obtain R.E.N.A.L., C-index, and CSA. Renal function was measured by 99mTc mercaptoacetyltriglycine (MAG3). We evaluated correlations between nephrometries and perioperative parameters, and comparatively analyzed different nephrometries to determine the predictive ability in the percent change of effective renal plasma flow of the affected kidney.

**Results:**

Three, two, and 35 patients received partial nephrectomy in open, laparoscopic, and robotic approaches, respectively. The median (IQR) tumor size was 3.13 (2.4) cm. The median (IQR) R.E.N.A.L., PADUA, C-index, and CSA scores were 7 (3), 8 (2), 2.01 (1.87), and 14.14 (19.25) cm^2^, respectively. Spearman correlation analysis showed that four nephrometries were correlated with each other. The strongest correlations were between CSA and C-index (coefficient: − 0.885, *p* < 0.001), followed by R.E.N.A.L. and PADUA (coefficient: 0.778, *p* < 0.001). Ischemia time was significantly correlated with R.EN.A.L. (coefficient: 0.35, *p* = 0.025), PADUA (coefficient: 0.42, *p* = 0.007), C-index (coefficient: − 0.45, *p* = 0.004), and CSA (coefficient: 0.41, *p* = 0.009). In multivariate analysis, PADUA significantly affected ischemia time (*p* = 0.04). The percent change in effective renal plasma flow (PCE) of the operated kidney was correlated with PADUA (coefficient: 0.48 *p* = 0.002), C-index (coefficient: − 0.74, *p* < 0.001), and CSA (coefficient: 0.75, *p* < 0.001). Only CSA and C-index independently affected PCE (both *p* < 0.05) in multivariate analysis. In ROC curve analysis, both C-index and CSA could predict 20% change in effective renal plasma flow (AUC: 0.91 vs 0.86, *p* = 0.2) of the affected kidney.

**Conclusions:**

We suggest using PADUA to evaluate surgical complexity and ischemia time. Regarding the accuracy of the prediction of post-operative ipsilateral renal function, both CSA and C-index outperformed R.E.N.A.L. and PADUA nephrometries.

## Background

Partial nephrectomy (PN) is the gold standard treatment for T1 renal tumors and even some T2 renal tumors in present clinical practice [1, 2]. Compared to radical nephrectomy, PN can decrease the incidence of renal dysfunction which may lower the incidence of non-cancer mortality [3, 4]. In order to improve rates of achieving of trifecta or pentafecta in PN, comprehensive evaluation of the tumor’s complexity is necessary before surgery. Therefore, using an adequate tool to evaluate the feasibility of PN and even predict the post-operative outcome is a critical issue in the treatment of localized renal tumors.

Various nephrometry systems have proposed to standardize the description and academic recording of renal tumors. The R.E.N.A.L. nephrometry system was the first to be proposed, followed by the PADUA classification system. These two systems use similar semi-quantitative anatomical factors and methodologies [5, 6]. In the contrast, C-index and mathematical tumor contact surface area (CSA) yield continuous variables which describe the geometric relationship between the tumor and kidney [7, 8]. These four nephrometry systems have been validated externally and they have all been associated with peri-operative outcomes [8–10].

Previous studies have tried to clarify the correlations between nephrometry systems and global functional outcomes assessed by estimated glomerular filtration rate (eGFR) [9, 11]. However inconsistencies and inaccuracies have been reported when using eGFR in patients undergoing PN because of compensatory hypertrophy of the contralateral kidney [12]. Split renal function assessed by radio-isotope scans has been shown to be more precise than eGFR to estimate renal function [13, 14]. In addition 99mTc mercaptoacetyltriglycine (MAG3) is preferred over 99mTc diethylene triamine penta-acetic acid (DTPA) in patients with impaired renal function.[15]. R.E.N.A.L. score and its individual constituents “R,E,N” have been reported to be relevant to ipsilateral post-operative renal function (IPRF) [16]. However, little is known about head-to-head comparisons of the predictive ability of IPRF using different nephrometries. In this study, we aimed to evaluate correlations between R.E.N.A.L, PADUA, C-index, and CSA and perioperative and renal functional outcomes assessed by radio-isotope scans.

## Methods

### Patients and data collection

After Institutional Review Board approval, we evaluated consecutive patients who underwent PN via open (OPN), laparoscopic (LPN), or robotic assisted (RPN) approaches for localized renal tumors between May 2013 and April 2017 at a tertiary referral center. We excluded patients with multiple renal tumors within one kidney, solitary kidneys, or end-stage kidney disease. The choice of surgical approach and resection and repair techniques were based on the surgeons’ expertise and the patients’ preference. All PN procedures were performed with the conventional on-clamp technique with clamping of both the renal artery and renal vein. All of the patients had pre-operative imaging with either computed tomography or magnetic resonance imaging. Cold ischemia was used only in OPN, and warm ischemia was used in both LPN and RPN. The patients’ demographics, clinical data, and imaging studies were obtained electronically and analyzed retrospectively.

Preoperative demographics data (sex, age, American Society of Anesthesiologists score, Charlson Comorbidity Index), perioperative outcomes (operative time, ischemia time, estimated blood loss, perioperative complications, length of hospitalization), and pathology features were recorded and evaluated. Nephrometry score including R.E.N.A.L., PADUA, C-index, CSA were obtained according to the original studies [5–8]. Global renal function was assessed according to eGFR. Split renal function represented by effective renal plasma flow (ERPF) was measured by 99mTc-MAG3 pre-operatively and 1 year after surgery. IPRF was reported as the absolute change of ERPF (ACE), and percent change of ERPF (PCE) of the operated kidney.

### Statistical analysis

Continuous variables are shown as median (IQR). Categorical variables, including those with a > 20% change of ERPF (PCE20) are shown as percentage. Spearman correlation analysis was used to evaluate the relationships among R.E.N.A.L. score, PADUA, C-index, and CSA and perioperative outcomes. Univariate and multivariate analyses of various clinical variables including nephrometries and PCE of the operated kidney were performed using linear regression models. The predive ability of nephrometries for PCE of the operated kidney was evaluated and compared using ROC curve analysis. All analyses was performed using SPSS v.22 (SPSS,Chicago,IL,USA), and a *P* value < 0.05 was considered to be statistically significant.

## Results

Three, two, and 35 patients received PN via open, laparoscopic, and robotic approaches, respectively. The median (IQR) tumor size was 3.13 (2.4) cm. The median (IQR) R.E.N.A.L., PADUA, C-index, and CSA were 7 (3), 8 (2), 2.01 (1.87), and 14.14 (19.25) cm^2^, respectively. The median (IQR) cold ischemia times and warm ischemia times were 28 (3) minutes and 20.5 (6.38) minutes, respectively (Table 1). The overall complication rate was 22.5%. Of the complications, four patients had urinary infections, two had pneumonia which was cured by antibiotics, two had anemia and received blood transfusions and 1 had a stress ulcer which was treated with a proton pump inhibitor. None of the patients had Clavien-Dindo grade 3 or higher complications. Pathological features demonstrated that renal cell carcinoma accounted for 57.5% of all tumors. Among them, 69.6% were pT1a, 21.7% were pT1b, and 8.7% were pT2 (Table 2).

**Table 1:** Demographic information of the study population

Variables	*N* = 40
Age, years	58 (11.25)
Male gender	23 (57.5)
ASA	2 (0)
CCI	0 (1.75)
Tumor size, cm	3.13 (2.4)
Depth of invasion, cm	1.6 (1.02)
RENAL score	7 (3)
PADUA	8 (2)
C-index	2.01 (1.87)
CSA, cm^2^	14.14 (19.25)
Minimal invasive surgery	37 (92.5)
Time from pre-operative ERPF to surgery, days	15 (8)
Time from surgery to post-operative ERPF, months	15 (1.5)

Data are expressed as median (IQR), or n (%)

*ASA* American Society of Anesthesiologists score, *CCI* Charlson Comorbidity Index, *CSA* Mathematical Contact Surface Area, *ERPF* effective renal plasma flow

**Table 2:** Perioperative features and change in renal function

Operative time, minutes	207.5 (101.75)
Ischemia time, minutes	21.02 (8.38)
Cold ischemia time, minutes	28 (3)
Warm ischemia time, minutes	20.5 (6.38)
EBL, mL	100 (237.5)
Complication	
Major, Clavien-Dindo grade 3 or more	0 (0)
Minor, Clavien-Dindo grade 2 or less	9 (22.5)
Length of stay, days	6 (1.75)
Pathological features	
RCC	23 (57.5)
T1a	16 (69.57)
T1b	5 (21.74)
T2	2 (8.7)
Oncocytoma	2 (5)
Angiomyolipoma	14 (35)
Carcinoid	1 (2.5)
Pre-operative	
Global eGFR, ml/min/1.73m^2^	94 (27)
ERPF of affected kidney, ml/min/1.73m^2^	187.08 (76.11)
Post-operative	
Global eGFR, ml/min/1.73m^2^	82 (31)
ERPF of affected kidney, ml/min/1.73m^2^	149.41 (169.45)
Functional change	
Percent change of eGFR	23.2 (12.51)
Absolute change of ERPF	47.65 (69.39)
Percent change of ERPF	22.16 (34.45)

Data are expressed as median (IQR), or n (%)

*EBL* estimated blood loss, *RCC* renal cell carcinoma, *eGFR* estimated glomerular filtration rate, *ERPF* effective renal plasma flow

The median (IQR) time from preoperative ERPF to surgery was 15 (8) days, and the median (IQR) time from surgery to postoperative ERPF was 15 (1.5) months. The median (IQR) ACE was 47.65 (69.39) ml/min/1.73 m^2^, and the median (IQR) PCE was 22.16 (34.45) % (Table 2). Spearman correlation analysis showed that four nephrometries were moderately to strongly correlated with each other. The strongest correlations were between CSA and C-index (coefficient: − 0.885, *p* < 0.001), followed by R.E.N.A.L. and PADUA (coefficient: 0.778, *p* < 0.001). Ischemia time was significantly correlated with R.EN.A.L. (coefficient: 0.35, *p* = 0.025), PADUA (coefficient:0.42, *p* = 0.007), C-index (coefficient: − 0.45, *p* = 0.004), and CSA (coefficient: 0.41, *p* = 0.009). Operative time was correlated with C-index (coefficient: − 0.34, *p* = 0.037) and CSA (coefficient: 0.37, *p* = 0.018), and PCE of the operated kidney was correlated with PADUA (coefficient: 0.48 *p* = 0.002), C-index (coefficient: − 0.74, *p* < 0.001), and CSA (coefficient: 0.75, *p* < 0.001). However weaker correlation coefficients for PCE/ ACE were noted in PADUA compared with C-index and CSA (Table 3).

**Table 3:** Correlation between nephrometries and peri-operative features

Variables	RENAL		PADUA		C-index		CSA	
	coefficient	*P*-value	coefficient	*P*-value	coefficient	*P*-value	coefficient	*P*-value
RENAL			0.778	< 0.001	−0.372	0.02	0.44	0.005
PADUA	0.778	< 0.001			− 0.622	< 0.001	0.647	< 0.001
C-index	− 0.372	0.02	− 0.622	< 0.001			−0.885	< 0.001
CSA (cm^2^)	0.44	0.005	0.647	< 0.001	−0.885	< 0.001		
EBL (ml)	0.179	0.269	0.082	0.616	−0.115	0.485	0.161	0.321
Operative time (minutes)	0.278	0.082	0.218	0.176	−0.336	0.037	0.373	0.018
Length of stay (days)	0.178	0.272	0.256	0.11	−0.187	0.254	0.107	0.511
Complication (Clavien-Dindo classification)	−0.137	0.399	0.075	0.647	−0.147	0.373	0.163	0.314
Ischemia time (minutes)	0.354	0.025	0.421	0.007	−0.453	0.004	0.409	0.009
Absolute change of ERPF (ml/min/1.73m^2^)	0.195	0.235	0.369	0.021	−0.687	< 0.001	0.680	< 0.001
Percent change of ERPF (%)	0.263	0.106	0.476	0.002	−0.740	< 0.001	0.747	< 0.001

*CSA* Mathematical Contact Surface Area, *EBL* estimated blood loss, *ERPF* effective renal plasma flow

In univariate analysis, operative time, R.E.N.A.L., PADUA, and C-index significantly affected ischemia time. However, only PADUA (*p* = 0.04) significantly affected ischemia time in multivariate analysis (Table 4). Ischemia time, PADUA, C-index, and CSA affected PCE of the operated kidney in univariate analysis, while only C-index (*p* = 0.03) and CSA (*p* = 0.005) influenced PCE of the operated kidney independently (Table 5). The predictive value for PCE20 of C-index and CSA were evaluated via ROC curve analysis, in which both C-index and CSA were equally able to predict the PCE20 (AUC: 0.91 vs 0.86, *p* = 0.2). The cut off values of PCE20 derived from Youden’s index were 2.11 for C-index and 10.37 cm^2^ for CSA.

**Table 4:** Regression analysis in ischemia time

Variables	Univariate			Multivariate analysis		
	B	(95% CI)	*P*-value	B	(95% CI)	*P*-value
Age	− 0.07	(−3,0.15)	0.52			
Sex	−1.45	(−6.2,3.3)	0.54			
ASA	−0.95	(−5.92,4.02)	0.7			
CCI	−0.3	(−2.11,1.5)	0.74			
Operative method	−2.14	(− 6.35,2.06)	0.31	0.5	(−4.14,5.15)	0.83
Operative time	0.04	(0.01,0.07)	0.03	0.02	(−0.01,0.06)	0.23
R.E.N.A.L.	1.69	(0.46,2.92)	0.009	−0.32	(−0.34,1.71)	0.75
PADUA	2.63	(1.27,34)	< 0.001	2.85	(0.09,5.62)	0.04
C-index	−1.6	(−3, −0.21)	0.03	−0.16	(− 3.12,0.8)	0.24
CSA	0.17	(−0.04,0.39)	0.11	−0.18	(0.47,0.11)	0.22

*ASA* American Society of Anesthesiologists score

*CCI* Charlson Comorbidity Index

*CSA* Mathematical Contact Surface Area

**Table 5:** Regression analysis in ERPF

Variables	Univariate analysis			Multivariate analysis		
	B	(95% CI)	*P*-value	B	(95% CI)	*P*-value
Age	−0.04	(− 0.77,0.69)	0.91			
Sex	−1.09	(− 16.76,14.59)	0.89			
ASA	3.55	(−12.4,19.49)	0.66			
CCI	−0.29	(−6.53,5.95)	0.93			
Operative method	−2.16	(−18.13,13.81)	0.79			
Ischemia time (minutes)	1.23	(0.22,2.17)	0.01	0.57	(−0.34,1.48)	0.21
Operative time (minutes)	0.02	(−0.1,1.35)	0.76			
Complication (Clavien-Dindo classification)	10.93	(−7.82,29.67)	0.25	−0.47	(−15.12,14.17)	0.95
EBL	0.02	(−0.02,0.05)	0.34	< 0.001	(−0.020.02)	1
R.E.N.A.L.	3.43	(−0.9,7.76)	0.12	0.78	(−4.46,6.18)	0.77
PADUA	6.21	(1.45,10.96)	0.01	−2.9	(−10.39,4.59)	0.44
C-index	−9.71	(−13.17, −6.25)	< 0.001	−5.14	(−9.8, −0.48)	0.03
CSA	1.57	(1.06,2.08)	< 0.001	1.1	(0.36,1.86)	0.005
ERPF of affected kidney (pre-operative)	0.03	(−0.12, 0.18)	0.71			

*ASA* American Society of Anesthesiologists score

*CCI* Charlson Comorbidity Index

*EBL* estimated blood loss

*CSA* Mathematical Contact Surface Area

*ERPF* effective renal plasma flow

## Discussion

In recent decades, a few studies have focused on factors impacting functional changes after PN, and identified both unmodifiable and modifiable factors [17]. The two most important modifiable factors, ischemia time and renal volume preservation, indicate the quality and quantity of the affected kidney, respectively [18]. The correlation between post-operative global renal function and different nephrometry systems varies from study to study [9, 11]. Furthermore, few head-to-head comparisons of the predictive ability of IPRF between different nephrometries have been reported. To the best of our knowledge, this is the first study to compare correlations between different nephrometries and IPRF. In this study, the four nephrometries were correlated with peri-operative outcomes and IPRF. In addition, we found that PADUA could significantly predict the ischemia time, while C-index and CSA independently affected IPRF.

The indication for PN has progressed from small renal masses to renal tumors < 7 cm in size [1, 2]. Contralateral kidney compensation becomes significant with greater parenchymal loss in patients with larger renal tumors [12]. Traditional models used to calculate eGFR according to serum creatinine have limitations such as estimation errors and inconsistencies, and therefore eGFR is suboptimal to assess functional outcomes [13]. Thus, assessing ipsilateral renal function using radio-isotope scans has become the standard evaluation method [1, 2]. Takagi et al. studied patients with small renal masses with a median size of 3.5 cm, which is similar to our study. In their study, the percent eGFR changes in operated and contralateral kidneys were − 24.4 and + 2.29%, respectively [12]. Other series have also reported that about 72–80% renal function preservation in the operated kidney [14]. The volume of renal preservation has been shown to be more important than ischemia time for long-term renal function in recent studies [17, 19].

Perioperative outcomes and different nephrometries have been highly correlated [9]. Borgmann et al. demonstrated that surgical complexity and trifecta of PN were better assessed by R.E.N.A.L score than C-index [20]. In the current study, PADUA nephrometry significantly affected ischemia time, while C-index and CSA did not. This may be because ischemia time reflects the surgical complexity, which involves the tumor location and sinus or collecting system invasion. In addition, it is better described by the comprehensive multiple anatomical factors included in PADUA. Recently Ficarra et al. proposed an updated version of PADUA, the Simplified PADUA REnal (SPARE) nephrometry, which exerts similar predictive ability of complication. Compared with CSA, SPARE nephrometry is not an independent predictor of renal function impairment [21]. It is possible that multiple factors with equal weights in the PADUA system may weaken the power in predicting IPRF.

Kwon et al. and Yoo et al. reported that R.E.N.A.L. was an independent predictor of IPRF as measured by 99mTc DTPA [22, 23]. In contrast to their study, R.E.N.A.L. score in our cohort was not a significant predictor of IPRF. The difference may be because the previous study treated the R.E.N.A.L. score as a binary variable and analyzed it with different ischemic types in linear multivariate analysis [22]. More importantly, the quality of 99mTc MAG3 images as used in our study has been reported to be superior to scans obtained using 99mTc DTPA in patients with diseased kidneys [15, 18]. A recent study further revealed that only the constituents of “R” and “E” in R.E.N.A.L nephrometry instead of total score were associate with ipsilateral renal function [16]. These two constituents are included in CSA and C-index.

Simmon et al. reported that C-index affected the nadir and late eGFR [24]. Samplaski et al. further reported that a C-index cut-off value < 2.5 increased the probability of a 30% decline in global renal function by 2.2-fold [25]. Another study reported that C-index outperformed R.E.N.A.L. score in predicting global renal function loss [10, 26]. To the best of our knowledge, this study is the first cohort study to evaluate the predictive ability of IPRF using C-index. Several potential reasons may explain the difference in cut-off value (2.5 vs 2.1) to predict renal functional loss. First, different methodologies were applied to yield cut-off values in the previous study [25]. Second, greater global function loss was reported in the previous study compared to in our cohort [25]. Third, split renal function is more sensitive than eGFR, so the cut-off value for the same percentage of functional reduction may be smaller.

**Fig. 1:**
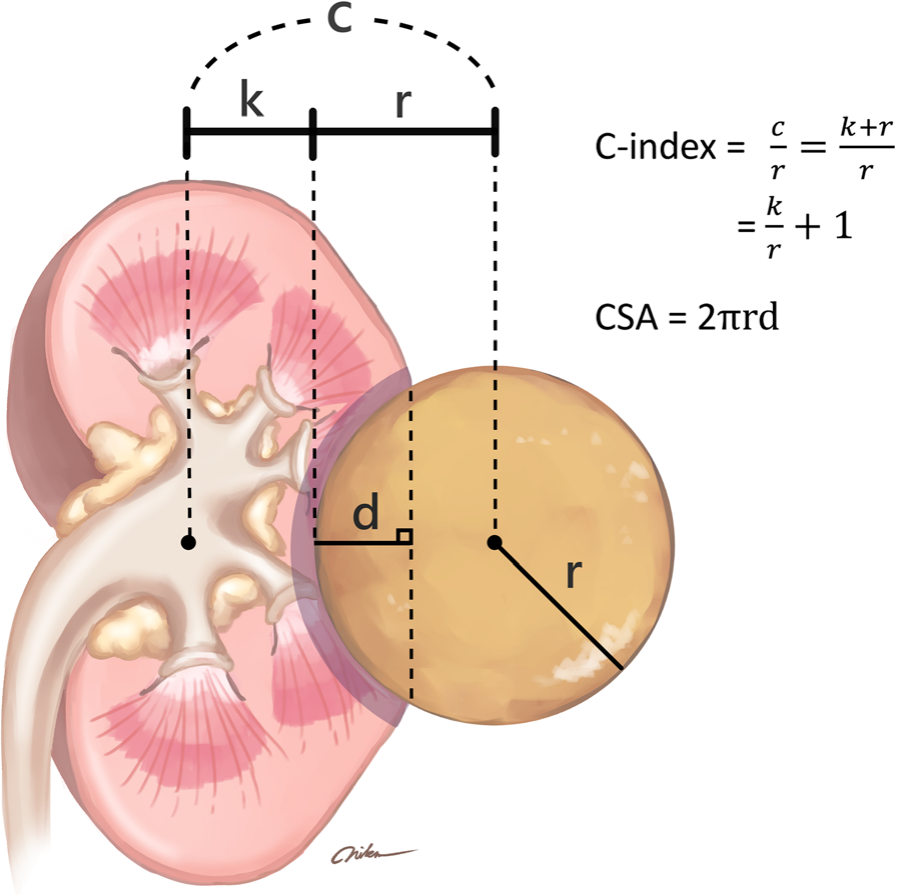
Geometric interplay between renal volume, C-index, and CSA; r: tumor radius, d: depth of invasion, k: nearest distance between tumor margin and kidney center; This figure is created by YPW

Both CSA and C-index described the degree of tumor invasion and had the common factor of tumor size. Moreover, depth(d) in CSA and distance(c) in C-index are very closely related. Our study further proved this concept because these two nephrometries were highly correlated (coefficient: − 0.885, *p* < 0.001). The impact of CSA and C- index on IPRF is supported by evidence showing that tumor size and depth are the most important prognostic factors of ipsilateral renal function [27]. The interplay between volume, CSA, and C-index is described in Fig. 1. The distance between tumor and kidney center (c) consists of tumor radius (r) and nearest distance between tumor margin and kidney center (k). Depth (d) implies the volume of resected renal parenchyma, and CSA was shown to be correlated with resected and ischemic renal volume in our previous study [28]. In addition, “k” implies the thickness of residual renal parenchyma, and C-index has been shown to be correlated with percent functional volume preservation [24]. Lee et al. found that CSA and C-index independently affected the percent reduction in renal cortical volume. [29] Therefore, both CSA and C-index were effective in predicting IPRF based on mathematical volume theory.

The strength of this study is that it is the first cohort study to compare ipsilateral functional outcomes among four nephrometries. In contrast to previous studies we provided more precise data derived from 99mTc MAG3 studies. Moreover, we directly compared the ability and provided cut-off values to predict functional changes for both CSA and C-index. There are also several limitations in this study. First, it was a retrospective design with a relatively small number of cases, and therefore selection bias was possible which may have resulted in confined tumor complexity and may be not representative the experience of other centers. However, the tumor complexity in our cohort was similar to previous studies [11, 20]. Although the follow-up time was only 1 year, Porpiglia et al. showed that ERPF remained stable 3 months after surgery [30]. Second, some of the renal tumors in our study were angiomyolipoma or other benign histology. However, the degree of global renal functional change in patients with angiomyolipoma after PN has shown to be similar to RCC [31]. Third, the PN technique such as enucleation and wedge resection was not unified. Therefore, further studies are needed to evaluate relationships among different nephrometries and pathologic or radiographic renal volume.

## Conclusions

Based on the results of this study and the different characteristics of each nephrometry systems, we suggest using PADUA nephrometry to evaluate surgical complexity and ischemia time. Regarding the precise prediction of IPRF, we recommend using both CSA and C-index, as this may have greater predictive ability than R.E.N.A.L. and PADUA nephrometries.

## Data Availability

The data supporting the conclusions are contained within the manuscript. The datasets used and analyzed during the current study are available from the corresponding author on reasonable request.

## Abbreviations

ACE: Absolute change of ERPF
CSA: Mathematical tumor contact surface area
DTPA: Diethylene triamine penta-acetic acid
eGFR: estimated glomerular filtration rates
ERPF: Effective renal plasma flow
IPRF: Ipsilateral post-operative renal function
LPN: Laparoscopic partial nephrectomy
MAG3: Tc-99 m mercaptoacetyltriglycine
OPN: Open partial nephrectomy
PCE: Percent change of ERPF
PN: Partial nephrectomy
RPN: Robotic-assisted laparoscopic partial nephrectom
